# Supplementary material for: Visualizing changes to US federal environmental agency websites, 2016–2020
Source: PLoS One. 2021 Feb 25;16(2):e0246450. doi: 10.1371/journal.pone.0246450 (PMC7906373; doi:10.1371/journal.pone.0246450)
Supplement: S1 File — (DOCX) [file pone.0246450.s001.docx]

1. [Data](https://github.com/edgi-govdata-archiving/web_monitoring_research/) files - list of URLs, list of key terms, term counts, and link counts: https://github.com/edgi-govdata-archiving/web_monitoring_research/tree/main/data
2. Python script for scraping terms and links: https://github.com/edgi-govdata-archiving/web_monitoring_research/blob/main/ctrl-f.py
3. Jupyter Notebook R script for producing visualizations: https://github.com/edgi-govdata-archiving/web_monitoring_research/blob/main/EDGI_TermAnalysis.ipynb
