# Supplementary material for: Visualizing changes to US federal environmental agency websites, 2016–2020
Source: PLoS One. 2021 Feb 25;16(2):e0246450. doi: 10.1371/journal.pone.0246450 (PMC7906373; doi:10.1371/journal.pone.0246450)
Supplement: Attachment — Submitted filename: reviewer response.docx [file pone.0246450.s002.docx]

**Reviewer 1 – Thank you for your constructive engagement with our manuscript!**

Major comments:

The abstract reports more substantial webpage changes at Cabinet level agencies and concludes that this is advancing an anti-science and deregulatory agenda. This is a large intellectual leap. If the authors would like to make the case for the connection between their findings and broader commentary about the agenda of the administration, there should be a larger discussion and documentation of that connection in the text. Alternatively, this language in the abstract could be adjusted to exclude this commentary on the administration’s agenda.

- We agree that this claim was too bold for the evidence at hand. We removed the assertion that the larger degree of information removal on more visible webpages evidenced the Trump anti-science agenda. Instead, we state that finding in the abstract, and in the body of the paper we discuss the congruency between those web actions and the Trump administration's efforts to influence public perception. We also changed from "explaining" to "analyzing" to acknowledge that we are offering hypotheses, not vetted explanations, based on our findings.

While the authors are limited to the timeframe of 2016-2020 given their data collection benefits, I think the manuscript could benefit from acknowledge of prior understanding of this issue. For example, how normal are these kinds of web changes under any administration? There is unlikely to be quantitative historical data on this but examples of individual cases or at least discussion of what we might expect of other administrations would be useful for readers. For example, under the Bush administration there were some inappropriate changes to agency science webpages, such as a case where inaccurate information falsely linking abortion to breast cancer. These kinds of cases could be illustrative of the historical context on these issues.

- Thank you for this very helpful suggestion! In the Framework section, we have pointed to other examples, e.g. Obama EPA's use of social media, and the Bush era web removals. We can also say, yes, this has happened before! And it is expected from any administration change. What's different is the removal of entire sites. [epa.gov/climatechange](http://epa.gov/climatechange) persisted across Clinton, Bush, and Obama admins.
- In addition, we have reiterated in our conclusion we acknowledge/point to the " lack (of) a framework for understanding how government websites change under different administrations and what standards these administrations are held to when they change their online content." We note that, thanks to a nonprofit organization (the Internet Archive), we know at least some resources that have persisted since the early days of federal websites. But, we have no systematic way of understanding what has changed until now.

Related to the above, such a discussion could also help orient readers to understand the universe of reasons that web text might be changed and the degrees of appropriateness of different kinds of changes (e.g. new political priorities of an administration, evolution of understanding on a scientific topic and evolving use of certain terms are appropriate reasons web text could change while censoring scientific language to serve a political agenda is not).

- Thank you for this suggestion! We have expanded on this in the framework section.

•	I’d suggest the authors strengthen the arguments for why web changes matter. For example, what can be said about public trust of the government, the public expectation of accurate communication from government entities, and the risks of irresponsible web changes? Is there an outsized impact on certain populations, such as the disability community who might rely on specific keywords, or Alaskan Native communities for whom the government may be one of the few localized sources of information on climate change? This could be strengthened in the introduction as well as in the discussion around line 661, where the authors could discuss reasons this work is valuable beyond government accountability. For example, the documentation of this work is invaluable for future administrations now that a methodology and characterization exists for a presidential first term. This is a huge contribution to understanding in our increasingly digital government world.

- We more thoroughly grounded our work in the e-government, information access, and government trust literature in the introduction to illustrate why federal website information, and changes to it, matters. The primary points are that public discourse is foundational to our democracy, trust in government has been fairly low for the last 50 years, and web-based information has been seen as a potential to rebuild public trust in government and be the conduit for enhancing information provision and enhancing democratic dialogue. However, that has not necessarily been borne out, and federal restrictions on information have been viewed unfavorably and had poor societal consequences. Thus, understanding recent changes to federal websites are important to understand their effects on society and environmental digital politics.
- In response to the outsized impact on specific populations - yes, we now discuss how web changes are impactful in socially uneven ways. For instance, while the EPA created an official archive of its English-language climate change pages, it did not do so for the Spanish-language versions, even further narrowing access to these resources along an axis of social inequality.

The authors operate under the frame that changes to lower-level pages are more likely to be self-censorship, thus, suggesting that changes to pages with shorter url’s are more likely to be directly political interference? I am not sure how much evidence there is to suggest this distinction. We’ve seen high levels of self-censorship broadly under this administration, even from fairly senior career staff. It seems plausible that changes to higher level pages are at least equally likely to be self-censorship. Alternatively, if the authors have examples or other evidence that there is direct political interference occurring at higher level pages, this could be stated to build their case.

- Thank you for noting this. Rather than asserting a hypothesis about political interference here, we’ve reframed our argument to be more thoroughly based in the data at hand, and note that language changes on higher level pages would be consistent with the most efficient way to alter public perception of an issue, and changes made on lower level pages would be consistent with a more thorough routing of information or self-censorship.

Minor comments:

I greatly appreciate the authors’ honest commentary about trying things that were unsuccessful and admitting they struggled in areas. I wish more scientific papers were written with this kind of honest and unfiltered commentary.

- Thank you!

I’d suggest a manuscript title that is more specific and descriptive about web changes specifically to draw relevant audiences.

- Visualizing Changes to US Federal Agency Websites, 2016-2020

Line 146: Please clarify that the topic is politically charged so as not to feed the narrative that it is scientifically controversial because it is not.

- We agree and have reworked this section to underscore the misinformation campaign.

Line 151: I would broaden this to clarify it is used by industry communications at large, not just lobbyists and not just oil.

- We expanded.

Line 157: I could not find this specific article, but based on the paraphrase, this does not seem like a good characterization of what is happening. No one is defined in climate change in terms of greenhouse gas emissions exclusively. Instead, this section should include reference to the decade-long coordinated misinformation campaign to sow doubt about climate change and the impact this has had on public understanding and polarization. I would recommend instead citing the works of Dan Kahan, Ed Maibach, or Robert Brulle on this topic.

- We agree with the suggestion that this section should be about the coordinated misinformation campaign and have revised accordingly, including through the reference of some of this material.

In the legal section, I might suggest referencing the public issue around DOI’s destruction of records a few years ago. It was a great example of the scientific and NGO community being very alarmed but the legal and logistical dimensions were not widely known and the incident may even have been routine. It was a good example of the lack of clarity around legal issues in this realm. <https://www.archives.gov/press/press-releases/nr20-43>

- We now include the surprising statistic that only 2-3% of agency records are actually preserved, and mention the opacity of the process for retention or destruction.

Line 400-401 could be rephrased for readability.

- We have rephrased the sentences here to better describe what is going on in the figure.

The authors discuss the Federal Register and GAO pages as being places that “climate change” increased and this is clear on Figure 6—which is a fantastic figure that is a tremendous contribution to public understanding on this issue. It might be worth noting the relevant context for these sites, i.e. the FR includes many regulatory rollbacks which necessarily reference climate change and other key terms in the context of rolling back actions taken under the Obama administration. The GAO fulfills requests from Congress, many of which are likely investigations and questions about the actions (and lack of actions) around climate change taken by the administration. These situations are different than discussion of climate change on executive branch agency pages, which are more driving on political agenda’s and executive branch policy priorities.

- Thank you for this suggestion! Indeed this is very relevant and useful context. We have revised our discussion in this part of the paper to account for these differences between FR and GAO and between those two and executive branch agencies.

In the recommendations, I’d suggest the authors accommodate situations where information may need to be deleted or updated because of evolving scientific understanding where the outdated information could be problematic to preserve in an accessible way online. There are many examples where this can happen (e.g. evolving COVID science or a chemical spill where water/air was safe but subsequently discovered to be unsafe to consume)

- Yes, thank you for noting this important point. We have included it in our recommendations.

Line 161: Do you have a citation that this is true? This would surprise me if there is evidence of it. It is not clear to me that government is a primary source. If this is not demonstrably true I would change to “one way climate change is communicated to the public...”

- Thank you! We have rephrased this accordingly.

Line 216: Is “industry oriented portrayal of environmental issues” something you need to be held accountable for? Arguably they are within their right to change portrayal of issues. And there were cases of the Obama administration being too industry oriented too (e.g. fracking and water quality study release). This is not problematic in the way that censorship is. There are many other features of this administrations treatment of webpages, I would suggest mentioning here instead of industry-oriented portrayal of environmental issues, such as neglect of science, misinformation on pages, misleading content, erasure of concern and products designed for marginalized groups, etc.

- We agree that industry-orientation is not problematic in the same way that censorship is, but we do think that it is problematic when it conflicts with science and public health needs, and when agencies frame their work as supporting industry more so than the general public. We have retained this but also made clear here the other issues..

When keywords are mentioned, please specify whether these are the authors’ chosen keywords or the keywords on government pages set by page editors.

- We clarified the way we derived our list of keywords. The terms we include in our analyses are the words that were most frequently changed in the website changes on which we had previously published.

I find Figure 4 challenging to understand and unintuitive. The 38% decrease seems be the key point, but I don’t see how this portrayal shows that or provides other information.

- Reviewer 2 also found figure 4 challenging to understand and unintuitive. It represents our attempt to summarize both absolute and relative changes to the mention of “climate change.” We have made some changes - including a legend and some helpful lines - that we think guide readers to the main message. Our goal here is twofold: 1) illustrate that not all changes have been removals; 2) however, most changes have been removals; 3) many changes have been full removals - this is indicated with the trendline. The diagonal trend shows complete removal of the term "climate change" from many pages. We struggled with how to communicate both absolute *and* relative changes (a page may only lose 3 mentions of climate change, but if there were only 3 mentions on the page to begin with, this is a full removal and worth emphasizing). We believe the changes we have made communicate this.

**Reviewer 2 - Thank you for your incisive read of our manuscript and the push to tighten its conceptualization.**

Although the paper tackles an interesting topic, I would not recommend its publication in its current form. My main issue with the paper as it currently stands is its unclear framing. While the title of this article is “Change and accountability in digital environmental governance,” under the section “Framework” (line 144), the subheadings of “Climate Change Communication” and “Legal History of Records-Keeping Laws” etc., do not directly address what the authors mean by digital environmental governance. If it is the conceptual underpinning of the paper, then the authors should introduce what they mean by digital environmental governance more prominently in the introduction. Instead, it is mentioned for the first time at Lines 91-93 at the end of that paragraph, as almost an afterthought (“In general, environmental policy is not manifested on the web or in other technologies as much as it is mediated by data accessibility, content, and quality, in what has been called ‘digital environmental governance.’”). Governance is quite a different concept than policy, which is a tool of governance. The authors are conflating several ideas in the introduction and it becomes difficult to understand exactly where they are situating this work and to what scholarly conversations/literature/disciplines they are seeking to contribute to.

As an example of the conceptual muddiness, in the abstract there is mention of environmental politics, in addition to the mentions of digital environmental governance and policy in the introduction, there is also mention of environmental communication, e-governance, laws and legal history, transparency and translation into accountability and justice. On p. 11 lines 278-288, there is also reference to critical data studies at the STS literature (science, technology and society), which is quite different from the literature on e-governance and digital environmental governance (and actually i would add more critical of the potential benefits of such approaches). There is also discussion of injustices, which is assumed a priori is the result of removal of information from websites but never really established (i.e., how are the authors defining injustice, and why does simple removal of information constitute injustice?). I would suggest that the authors make clear at the outset the conceptual framing of the paper and tighten the introduction/background so that so many different terms, disciplinary angles, concepts are not at odds with each other or confusing the reader as to the article’s main point. The organization of the introduction can then be made clearer, but as it now stands, the authors need to make clear what they mean by digital environmental governance and how digital record keeping such as websites relates to “change and accountability in digital environmental governance,” which has scholarship and meanings beyond simply information and its removal on websites (see: Soma, K., MacDonald, B. H., Termeer, C. J., & Opdam, P. (2016). Introduction article: informational governance and environmental sustainability. Current Opinion in Environmental Sustainability, 18, 131-139. for an introduction).

- We greatly appreciate this comment as it has helped us clarify that our aim with this paper is to describe analytic methods not define a new term “digital environmental governance”. In this paper, our focus is on describing methods to document and analyze changes to federal environmental agency websites that are mostly automated, quantitative, summarizable, and visually interpretable (with broader application to other kinds of websites). Understanding/interpreting the meaning or importance of such changes involves analyzing how webpages mediate public understanding of policy and policy issues like climate change a thorough discussion of which is beyond the scope of this paper. Based on these very helpful suggestions, we decided we did not need to muddy the waters by naming something “digital environmental governance” (or digital environmental X). We have removed this from the paper. Instead, we more concretely engage the literature relevant to understanding web changes, posit our contribution as a methods paper (documenting, visualizing, and analyzing changes at scale), and do not attempt to invent a new concept. These changes are reflected in the following ways:
  - Removal of “digital environmental governance” from the title
  - Removal of this from the introduction, replaced by a more concrete discussion of the issue. Elsewhere in the introduction we also more clearly state what this paper is not.
  - More structure to the framework section to illustrate our key concerns in web changes, and the gaps and limitations in such work.
  - This includes removing work on critical data studies, which indeed did muddy the waters.
  - As well as removing questions of justice in this section that are not fully fleshed out in the results or discussion.
  - More fully returning to the framework in the discussion, noting more concretely how our work squares with the literature.

Methods - While I think the data the authors collected is impressive and worthy of analysis, the methods primarily focus on word count changes (i.e., more mentions of a word in one period versus the other). But the authors authors fail in the introduction and lack of conceptual framework/theory to explain WHY changes of word counts or change in language actually LEAD to differences in digital environmental governance. Is the theory that, when words are removed, an agency’s ability to govern climate change is diminished? Or if citizens no longer see climate change-related language on a government web page, they take less action themselves on climate change, or demand less action on the part of government agencies? or the removal of language is somehow associated with poor governance outcomes or processes? This theoretical underpinning and linkage is completely absent, and so the Methods cannot be evaluated appropriately.

- This is now addressed in intro, and elaborated on in theory and in results. For us, changes matter because “if citizens no longer see climate change-related language on a government web page, they take less action themselves on climate change, or demand less action on the part of government agencies”...*and* because ...”the removal of language is associated with poor governance outcomes or processes.” With respect to the latter, the website explains what the agency is doing. Removing language changes the explanation of what they are doing.

For example, in lines 323-324, the authors refer to methods “term analysis” and “link analysis” - are these common methods in text analysis? Can they include some references/citations for readers to better understand whether these are established and appropriate methods?

- We have altered our use of the terminology to match existing approaches. By term analysis we were referring to the widely used “content analysis” approach in the social sciences (which counts words). We have provided sources there. By “link analysis” we were referring to network analysis. For clarity, we have replaced term analysis with content analysis and link analysis with network analysis. Additionally, we reiterate our visualization methods are rooted in exploratory data analysis and have tried to state this more upfront.
- In addition, we have tried to clarify our approach by using the term “techniques” instead of “methods” as, yes, these visualizations reflect techniques or tools more than methods much less methodologies. We have also removed reference to exploratory data analysis - although it informs our approach, it is not the approach itself and reference to it may muddy the waters more than anything. The kinds of bar charts, dot plots, scatter plots, and small multiples we use in this paper are well-known outside of EDA.

Why did the authors not consider natural language processing techniques, particularly unsupervised techniques like topic analysis (see LDA or STM techniques), to examine changes in topics rather than in self-selected individual terms (more on that in the specific comments below).

- While advanced forms of topic and text analysis would be very interesting paths to pursue for further research, as we have tried to reiterate in these revisions, a primary goal of ours is to have a straightforward, computational approach that allows audiences to visually interpret data at scale. In the conclusion, we now highlight work that we think needs to be done by others underscored by our findings, including these kinds of approaches.

The authors also claim in the abstract to formulating new methods to “visualize and assess the alteration of websites,” although in looking through the figures, they seem like pretty standard data visualizations to me. Stacked bar plots, scatter plots, also the “directionality” scatterplots - these are already existing data visualization methods. So what’s new here? Additionally, data visualization is not really a research method. It is a research tool. I’m not saying there’s no value in descriptive analysis - i’m a strong believer in descriptive data analysis - but it needs to be clear how the method links to the conceptual theory.

- Our point is certainly not that they are entirely new methods - indeed they are quite standard - but new methods when it comes “to visualize and assess the alteration of websites....” We have emphasized a point originally made in the framework section: “Many of these evaluations have been agency- or website-specific, intimately characterizing web content and illustrating the practices by which it is altered and maintained, but comprehensive analyses are also needed.”

I think the authors would provide a much more compelling analysis if they went back to the conceptual drawing board and spelled exactly what the theory/framework is and HOW it relates to digital environmental governance. And then ask what kinds of data would help support/illustrate that theory. And then show data visualizations that then illustrate what they find. There is so much going on in these methods and it’s not clear to what end. There’s network analysis, word counting, “small multiples” (not sure what that means as there wasn’t a citation), institutional/agency positioning….

- There was a citation. The PLOS One citation format looks like this: (57). The number refers to its place in the references list. It’s confusing though because it reads as if it were our own indication of some count of something.

For the figures, many need a legend and they are not consistent - some provide the data in raw counts, some of them in percent change. More detailed comments below.

- We have added legends to the figures.
- It’s not clear to us why the figures would need to be consistent in the sense of raw counts vs percent change. We have tried to be clear why it can be useful to have both pieces of information. It is true that switching between the two can be confusing; we believe the legends clarify.

Other methodological points:

Lines 346-349: how did the authors choose the climate-change related words? It’s not clear how they documented change in EDGI’s 35 published reports, etc., and why/how.

- We identified the most frequently changed words observed in those reports to serve as keywords for the content analysis. Further detail on our weekly website monitoring is available from those reports themselves. We use open source tools we designed ourselves to visualize text and other changes (these are now implemented on the Internet Archive’s Wayback Machine too). Of course, not every change is significant or worth writing a report about. At our weekly meetings we discuss the changes we see and decide whether to pursue a report. We believe a full discussion of this dimension of our web monitoring is beyond the scope of this particular paper.

Lines 367-371: It’s hard to tell what’s going on in the network analysis/Fig. 3. It seems that there are almost even numbers of blue/red links, so equal numbers of links being added and removed. Did you calculate any network statistics to summarize the types of edges? I know it’s probably unwieldy to label all nodes, but is it possible label some of the nodes so we have an idea of which agencies these websites refer to? Can you explain the red cluster to the right?

- The figure is already partially labeled, but we have made some additional changes here in order to clarify what is going on. The confusion may stem from the fact that epa.gov/climatechange is located within the main cluster of nodes since it is was a navigation menu link, but subpages in the directory did not have direct connections to many other EPA pages and these were separated out into the red cluster at the right. To be clear, the red cluster at the right is epa.gov/climatechange pages, but our label for epa.gov/climatechange was at its position in the central cluster; hence, the confusion. We have adjusted accordingly and added several more labels to the nodes.
- We did not calculate network statistics for the purposes of clarity in this descriptive analysis but that could be a next step. However, we do report basic numbers of edges. Indeed, many new links were added but these were mainly to open data and legal disclaimer websites (see above).

Fig. 4 is really difficult to interpret. I’m not sure ‘counts’ is the way to present the x-axis. I wonder if you could just show year on x-axis and just show percentage change in use of climate change. It’s not clear what your hypothesis or theory here is - is it that websites that originally had more references to climate change now have fewer references compared to those that mentioned climate change less? I think you need some way of normalizing the presentation of this data. Without a legend that indicates what numbers the gradients refer to, it’s extremely difficult to understand what is being presented here.

- We have added a legend. Opacity = number of pages with that use of “climate change” in 2016 (x-axis) and change to 2020 (y-axis). Red = removals, blue = additions.
- It is not clear what year on the x-axis would mean, as we only have one timeframe differential (2016-2020), so there would only be one x-axis value (and only one y-axis value too).
- Our goal here is to: 1) illustrate that not all changes have been removals; 2) however, most changes have been removals; 3) many changes have been full removals - this is indicated with the trendline. We struggled with how to communicate both absolute *and* relative changes (a page may only lose 3 mentions of climate change, but if there were only 3 mentions on the page to begin with, this is a full removal and worth emphasizing). We believe the changes we have made communicate this.

I take the most issue with the “climate shifts” analysis. the authors need to provide stronger methodological framing for their choice in word substitutions. There is a lot of evidence that shifts away from directly referring to climate change as climate change in a US context is not due to weakening or avoiding climate action, but the opposite. i’d argue that sustainability and resilience are actually distinct or different aspects of climate change and not necessarily weakening or signalling retreat of climate change. ie referring to climate emissions as “carbon pollution” or “climate pollution” to signal to constituents that that carbon is actually a negative externality when Obama’s team was trying to get the Clean Power Plan passed through Congress the EPA specifically didn’t use “climate change” because of the politicization of the term. they therefore referred to the plan as the “Clean Power Plan” to try to secure more support. So simply shifting terminology does not necessarily signal weakening or moving away from climate action - in a complicated political context like the US, politicians often reframe climate policy in different terminology to secure support (i.e., cap and trade instead of a carbon tax because of the palatability of market-based mechanisms).

- Sustainability has a much broader scope than climate change, even as the two do intersect. We don’t disagree with the notion that rhetoric changes can signal a retreat from climate change action. Indeed, as we evidence elsewhere in the paper, they may enable agency staff to “fly under the radar” of higher ups. Our point here is simply that as a reader, seeing resilience instead of climate change implies different things, just as cap and trade may be more palatable than “tax” but is actually a different thing.

if the authors are going to continue with this analysis of “small multiples” they need to completely reframe the rationale given my points above. It’s not clear at all why the authors are framing these terms as “either-or” or inherently at odds. in fact, resilience as a term in the climate development community has picked up broader support in the last few years as a way to be inclusive and comprehensive in climate change policy, to incorporate not just considerations of mitigation but adaptation and future-proofing. So where is the evidence/foundation for its use as a means of avoiding/shirking climate change action?

- There is significant research making the point that resilience is a relatively vague term - indeed, that may be precisely why it is gaining in use, perhaps it enables more work to actually get done on climate change. That is probably why CRWU switched from Climate Ready to Creating Resilient Water Utilities. We make no assertion in this paper about why we see it. But it is not to say that switching to resilience - while it may facilitate some forms of action - does not have side effects. Research has illustrated some of the many limits of the notion of resilience - how the question “who is to be resilient to what?” is often not addressed. Indeed, resilience is not a suitable synonym for climate change, though that is how we have seen it used in some cases by US federal environmental agencies. It only describes (poorly) our goal in responding to climate change. It does not actually describe what climate change actually is (or, importantly, how to mitigate rather than recover from its impacts).

again raw counts are not useful - would either change to percent change or some other type of normalization (percentage of total text, etc.).

- We disagree. Raw counts can be useful. There is a big difference between seeing one mention on a page and 100. If all mentions were removed, the percent change metric would show them as similar changes when clearly the magnitude of one is greater than the other. Of course, percent change is important too (and percentage of total text, admittedly). That is precisely why we have visualized in several different ways - to account for both absolute and relative changes.

i don’t understand why there are 4 of these plots. the caption refers to quadrants but the quadrants aren’t labeled.

- We have revised the figure to better show that it consists of four smaller subplots (small multiples), which previously were “quadrants”.

agency analysis - how were the agencies selected?

- We have indicated in the Methods and Materials section how our coverage of federal environmental agencies came to be.

what’s the theory regarding ‘mentions’ of climate change and ‘digital environmental governance’? again this links back to the lack of a clear conceptual framework. Just mentioning climate change doesn’t translate into automatic digital environmental governance of the issue. User experience/confrontation of the word climate change on websites also doesn’t necessarily enhance governance of an issue - what’s the theory here?

- We have dropped the notion of “digital environmental governance” for the reasons explained above and instead more explicitly spelled out the reasons why coming across (or not) a word on a government website would matter - it shapes trust and certainty around an issue, as well as awareness of what government is doing about it (or not). These websites are trusted sources of info and while perhaps not as important of a source of climate information as the media, still important. All this situates our study in existing work on climate change communication (especially industry efforts to manufacture doubt and controversy) and e-government.

Fig. 6: i don’t understand the scale? why not just present a bar chart like i suggested for fig. 4?

- It is useful to have two pieces of information: 1. how often a page is/was mentioned across an agency's site 2. the change (below the line = less often mentioned in 2020). We have added more context (legends and axis ticks) to the figures that should help.

Fig. 8 - what does the size of the points refer to? it’s not explained in the legend.

- We added a legend. Circle size indicates the number of pages experiencing that degree of change.

Fig. 10 - need some sort of legend or glossary to spell out which agencies are being referred to.

- The agency acronyms are spelled out in the previous table.

Discussion/conclusion - I didn’t go through these sections in much detail because they mainly repeated the results. I think the authors would have a much richer discussion if they could link back their analysis/results to a conceptual grounding and reflect on how their results add to other similar studies or analysis.

- We have more directly linked results back to a conceptual grounding and have reflected on how they add to other similar analysis.

Other more specific/minor details - I’m not sure why FIg.1 is necessary. it’s clear from the text in Materials and Methods what the time period analyzed was and when the Obama/Trump Administrations occurred.

- There are some important nuances in our selection of time period, because some periods have better or more appropriate records than others (e.g. summer 2016 vs fall 2016). However, since it is clear from the text, we will remove the chart.

Fig. 2 - why did the authors exclude information in the navigation pages?

- Because these are not particularly meaningful mentions of terms. They are not used in any context (i.e. in a sentence) and they are repeated across an agency’s entire website, so their count would be inflated if we looked at these parts of each page. We have included this explanation in the text.

line 75: should define what is meant by “federal webscape” for those unfamiliar with the term.

- Revised for clarity: federal websites.

Comment 1: Line 366: This sub-section should also discuss the key links/pages that have been added to provide a more complete picture of the changes.

- We had this in a previous version of the manuscript and have brought it back in.

Comment 2: Line 394: Text should read Fig. 3 instead of Fig. 1

- Fixed.

Comment 3: Line 413: Again, I am not sure about this approach of examining web pages in isolation. I understand that this is a purposive sample, but -- even so -- it might be more pertinent to examine changes in the overall counts of terms between 2016 and 2020.

- We examine changes in overall counts in other figures. We have tried to clarify why we would be interested in both absolute and relative changes. In addition, we have tried to clarify why the use per page metric matters: it provides a sense of how likely a user would be to come across “climate change” while navigating an agency’s website.

Also, the authors might want to refer to Figure 1.3 on the following webpage for one approach to visualizing change in term counts/proportions: <https://www.tidytextmining.com/tidytext.html#tidyausten>.

- Figure 1.3 on that webpage is exactly what we do in our own Figure 6 (now 5).

Comment 4: Line 451: I understand that the authors might primarily want to illustrate the use of their method, but the rationale for comparison of "climate change" and "resilience" (only) is unclear. An overview of terms whose counts/proportions have changed the most would be useful before presenting this specific comparison.

- We have clarified our rationale for comparing climate change and resilience (beyond just, we need something as illustration for our method). The shift was very present in our weekly monitoring reports, and it is something for which there is a theoretical basis for examination. It is not our goal to assert any explanation for why there may be a shift from climate change to resilience (be it for agency staff to fly under the radar or to avoid the currently polarized connotation of the term “climate change”), simply that it is consequential. A more data-driven analysis - of which counts/proportions have changed the most - would be interesting but it is beyond the scope of this paper.

Comment 5: Line 487: I think this analysis should be modified to take page deletions into account, since those affect the users' online experience as well.

- This is a wonderful suggestion, but: 1) to some extent is already factored into the analysis; 2) not doing it makes our analysis more conservative.
- 1 - There are some pages, like epa.gov/climatechange that weren’t, from a technical perspective, deleted. Instead, they were “removed” from public access and a “redirect” was set up. Now when users try to access them, they are pointed to a new page, one that the Internet Archive’s Wayback Machine is archiving and that we *can* scrape. Many of the complete removals of key words like “climate change” (as evidence in figure 4) happened on this kind of “redirected” page. This is also true even for *some* deleted pages depending on the agency. For instance, deleted EPA pages redirect to a landing page that we can scrape and count.
- 2 - However, by not examining pages that were live in 2016 but deleted by 2020, our analysis of the scope of changes is conservative. There may be pages that we ended up excluding that would add more “removals” to our summary, such that the percent of mentions would be even greater (e.g. greater than 38% in the case of “climate change.”) We have tried to emphasize the conservative nature of our approach in these revisions.
